# Prevalence of Intestinal Protozoa Infections and Associated Risk Factors among Schoolchildren in Sanandaj City, Iran

**Published:** 2017

**Authors:** Pegah BAHMANI, Afshin MALEKI, Shahram SADEGHI, Behzad SHAHMORADI, Esmaeil GHAHREMANI

**Affiliations:** 1. Research Committee, Kurdistan University of Medical Sciences, Sanandaj, Iran; 2. Kurdistan Environmental Health Research Center, Kurdistan University of Medical Sciences, Sanandaj, Iran

**Keywords:** Intestinal protozoa, Prevalence, Risk factors, Schoolchildren, Iran

## Abstract

**Background:**

Intestinal parasites are still a serious public health problem in the world, especially in developing countries. This study aimed to assess the prevalence of intestinal protozoa infections and associated risk factors among schoolchildren in Sanandaj City, Iran.

**Methods:**

This cross-sectional study involving 400 schoolchildren was carried out in 2015. Each student was selected using systematic random sampling method. Questionnaire and observation were used to identify possible risk factors. Fresh stool samples were observed using formal-ether concentration method.

**Results:**

Five species of intestinal protozoa were identified with an overall prevalence of 42.3%. No cases of helminthes infection were detected. The predominant protozoa were *Blastocys hominis* (21.3%) and *Entamoeba coli* (4.5%). Overall, 143 (35.9%) had single infections and 26 (6.4%) were infected with more than one intestinal protozoa, in which 23 (5.9%) had double intestinal protozoa infections and 3 (0.5%) had triple infections. A significant relationship was observed between intestinal protozoa infection with economic status, water resources for drinking uses, and the methods of washing vegetables (*P*<0.05).

**Conclusion:**

Education programs on students and their families should be implemented for the prevention and control of protozoa infections in the study area.

## Introduction

The prevalence rate of the intestinal parasites is high around the world, especially in developing countries. According to WHO, three billion people are infected; out of which, about 450 million are infected as result of these infections (1, 2). These infections are often ignored until severing or chronic complications are observed because many of them are usually asymptomatic or manifest only mild symptom (3). Intestinal parasitic infections (IPIs) occur in both rural and urban population, especially in school age children due to their habit of playing or handling infected soils, eating with soiled hands, unhygienic toilet practices, and ingestion of contaminated food, water or soils (4, 5). Apart from causing morbidity and mortality, these infections can cause iron deficiency, retardation of growth, energy malnutrition, and low education performance of school-children (6, 7).

There are several environmental and socioeconomic factors contributed to the presence of IPIs in children. Among them are lack of education, poverty, poor sanitary conditions, the absence of potable water, inadequate disposal of excreta, and poor housing facilities (8, 9). In spite of remarkable development in medical science in recent years, the IPIs remain a serious health issue in developing countries (10). The environmental, geographical, and socio-economic factors are responsible for spatial distribution and prevalence of various species of IPIs in world (1, 11).

Compared with other age groups, school-children have the highest morality rate because of IPIs (12). Treating schoolchildren alone can prevent the rate of IPIs by about 70% in the whole population (13). Like in other developing countries, IPIs are a major health problem in Iran. The prevalence rate of IPIs among primary schoolchildren was 18.4% in Tehran, Iran (12). Therefore, study on the prevalence of various IPIs is required for not only planning and implementation of appropriate control programs but also to predict risk factors for communities under consideration (14).

Although several studies were carried out on the distribution and prevalence of IPIs in Iran, still there are several localities lacking epidemiological information on IPIs. Sanandaj City, located in northwestern Iran, is one of such localities without any report in this regard. Thus, this study aimed to assess the prevalence of intestinal protozoa infections and associated risk factors among schoolchildren in Sanandaj, Iran.

## Materials and Methods

### Study design and area

This cross-sectional study was carried out between Jan 5 to Feb 30, 2015, in Sanandaj City, northwestern Iran. The city height from sea level is 1450 to 1538 m. The average annual temperature ranges from −13.5 to 44 °C and the average annual rainfall is 3497 mm (15). The study population consists of 400 schoolchildren including all age groups of both sexes in primary and secondary schools from urban and rural settings. To select the study subjects, the students were first classified according to their educational level (grade 1 to 9) and they were taken from each class category by systematic random sampling using class roster as a sampling frame.

Prior to stool sample collection, preliminarily meeting was held up with the directors of the schools and head of healthcare centers and the outline of the project was explained. Then both schoolchildren and their parents were asked for verbal consent.

The day before sampling, special bags were distributed to the parents, with an information sheet explaining the procedure of sample collection, a structured questionnaire, along with a stool specimen container with toilet tissue paper. The questionnaire questions were structured based on possible risk factors (Table 1–3). Personal characteristics of students including age, sex, and educational grade were recorded. The following morning, the bags and the specimens were collected and were examined in a research laboratory of Kurdistan University of Medical Sciences, Iran. A part of each individual stool sample was processed using formal-ether concentration method and was examined under microscopic diagnoses to identify parasites.

**Table 1:** Overall prevalence of intestinal protozoa infections in relation to sex, age groups, residence, and school of children in Sanandaj City, Iran

**Characteristic**		**No of examined (%)**	**Positive (%)**	**Negative (%)**	**X^2^(*P*-value)**
Sex	Male	320 (80)	136 (42.5)	184 (57.5)	0.41 (0.9)
	Female	80 (20)	33 (41.3)	47 (58.8)	
Age (yr)	7–9	39 (9.8)	15 (38.5)	24 (61.5)	0.88 (0.64)
	10–12	154 (38.5)	62 (40.3)	92 (59.7)	
	13–15	207 (51.8)	92 (44.4)	115 (55.6)	
Residence	Urban	190 (47.5)	74 (38.9)	116 (61.1)	1.6 (0.22)
	Rural	210 (52.5)	95 (45.2)	115 (54.8)	
School of students	Primary	200 (50)	79 (39.5)	121 (60.5)	1.24 (0.31)
	Secondary	200 (50)	90 (45)	110 (55)	
Total		400	169 (42.3)	231 (57.8)	

**Table 2:** Prevalence of intestinal protozoa infections among age groups, sex, residence, and school in Sanandaj City, Iran

**Intestinal protozoa**		**No. of infected (%)**	**Age (yr)**			**Sex**		**Residence**		**School**	
			7–9	10–12	13–15	Male	Female	Urban	Rural	Primary	Secondary
	*B. hominis*	85 (21.3)	12 (30.8)	30 (19.5)	43 (20.8)	66 (20.6)	19 (23.8)	46 (24.2)	39 (18.6)	41 (19.3)	44 (28.6)
	*E. coli*	18 (4.5)	1 (2.6)	4 (2.6)	13 (6.3)	11 (3.4)	7 (8.8)	6 (3.2)	12 (5.7)	6 (3.2)	12 (5.1)
Single infection	*E.nana*	15 (3.8)	0.0	10 (6.5)	5 (2.4)	12 (3.8)	3 (3.8)	3 (1.6)	12 (5.7)	10 (5.2)	5 (2.1)
	*G.lamblia*	14 (3.5)	1 (2.6)	5 (3.2)	8 (3.9)	13 (4.1)	1 (1.3)	6 (3.2)	8 (3.8)	6 (3.4)	8 (3.9)
	*I. buetschlii*	11 (2.8)	0.0	5 (3.2)	6 (2.9)	9 (2.8)	2 (2.5)	3 (1.6)	8 (3.8)	6 (3.2)	5 (1.6)
	*E. hystolitica*	0.0	0.0	0.0	0.0	0.0	0.0	0.0	0.0	0.0	0.0
Multiple infection		26 (6.4)	1 (2.5)	8 (5.3)	17 (8.1)	25 (7.8)	1 (1.1)	10 (5.1)	16 (7.6)	10 (5.2)	16 (3.7)
Total		169 (42.3)	15 (38.5)	62 (40.3)	92 (44.4)	136 (42.5)	33 (41.3)	74 (38.9)	95 (45.2)	79 (39.5)	90 (45)

**Table 3:** Univariate logistic regression analysis for risk factors potentially associated with intestinal protozoa infections among Sanandaj City, Iran

**Risk factors**		**Positive (%)**	**Negative (%)**	**Crude odd ratio, 95% CI, *P*-value**
Economic status	Weak	24(64.9)	13(35.1)	0.31, 0.13–0.72, 0.007
	Average	94 (42.2)	129 (57.8)	0.43, 0.19–0.94, 0.03
	Good	51 (36.4)	89 (63.6)	1.00
Source of water for drinking	Tap water	105 (40.6)	157 (59.4)	0.79, 0.29–2.1, 0.64
	Wells	88 (67.0)	30 (33.0)	1.13, 1.37–3.4, 0.04
	Fountains	9 (45.0)	11 (55.0)	1.00
Method of washing vegetables	Water alone	184(63)	58(37)	2.05, 1.12–2.04, 0.03
	With added some substance	105(23)	53(77)	1
Hand washing after toilet	No	0.0	3(100)	0.0, 0.0, 0.99
	Water only	12 (40.0)	18 (60.0)	0.69, 0.3–1.58. 0.38
	Water and soap	157 (42.8)	210 (57.2)	1.00
Fathers educational status	Illiterate	16 (50.0)	16 (50.0)	0.89, 0.18–4.4, 0.89
	Grade 1–6	55 (46.2)	64 (53.8)	1.18, 0.34–4.06, 0.79
	Grade 7–9	35 (43.8)	45 (56.3)	1.12, 0.29–4.3, 0.86
	Grade 10–12	41 (41.6)	51 (58.4)	1.05, 0.29–3.8, 0.93
	Above diploma	22 (28.6)	55 (71.4)	1.00
Mother educational status	Illiterate	32 (50.8)	31 (49.2)	0.92, 0.37–2.3, 0.85
	Grade 1–6	62 (41.1)	89 (58.9)	0.66, 0.3–1.4, 0.31
	Grade 7–9	27 (39.0)	33 (61.0)	0.89, 0.37–2.1, 0.8
	Grade 10–12	29 (35.4)	41 (64.6)	0.84, 0.37–1.9, 0.69
	Above diploma	19 (33.9)	37 (66.1)	1.00
Occupation of parents	Merchants	123 (43.6)	160 (56.3)	1.07, 0.3–3.8, 0.85
	Government employees	25 (34.2)	48 (65.8)	1.05, 0.29–3.8, 0.93
	Farmers	10 (56.3)	7 (43.8)	1.42, 0.28–7.06, 0.66
	Unemployed	6 (42.9)	8 (57.1)	0.89, 0.18–4.4, 0.89
	Others	5 (38.5)	8 (61.5)	1.00
Grade of students	1–6	79 (39.5)	121 (60.5)	0.79, 0.5–1.23, 0.31
	7–9	90 (45.0)	110 (55.0)	1.00

### Statistical analysis

The date collected using questionnaires and parasitological examinations were analyzed using SPSS Ver. 20 (Chicago, IL, USA) statistical software. Chi-square (X^2^) test was used to assess relationship between risk factors and prevalence of IPIs. Crude Odds Ratio (COR) and 95% confidence interval (CI) were performed to determine the strength of the relationship between infection and risk factors. A *P*-value less than 0.05 were considered statistically significant.

## Results

### Study population demographic characteristics

Of 400 students participated, 320 (80%) were males. The mean age of the study subject was 12.3 yr old (range 7–15 yr). According to their residence, 190 (47.5%) were from urban and 210 (52.5%) schoolchildren were from rural areas. The study population consisted of students from primary and secondary schools with the same percent of 200 (50%) (Table 1).

### Prevalence of intestinal parasites

**Fig. 1:**
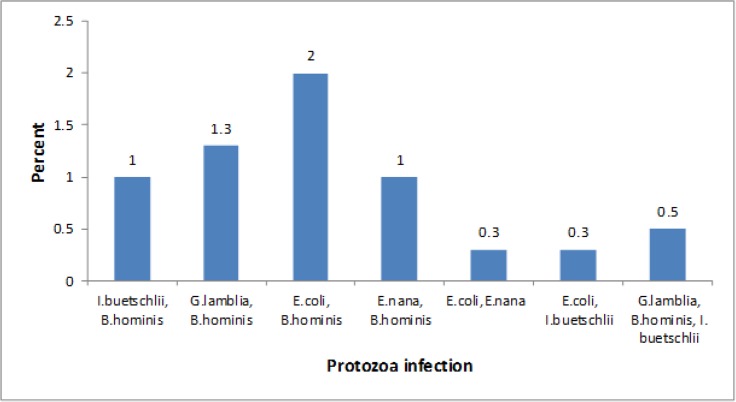
Prevalence of double and triple protozoa infections among schoolchildren Sanandaj City, Iran

Table 1 summarizes the distribution of parasite infection among primary and secondary schoolchildren in urban and rural areas. Out of the 400 stool samples examined, 169 (42.3%) were positive for one or more of intestinal protozoa. In total, out of 10 parasite species studied (four species of helminthes and six species of protozoa), only five species of protozoa were detected. Among protozoa, 136 (42.5%) males and 33 (41.3%) females were positive for one or more protozoa infections. 74 (38.9%) urban schoolchildren and 95 (45.2%) rural schoolchildren were infected with one or more than one intestinal protozoa. However, there was no significant difference (*P*>0.05) (Table 1). Five major intestinal protozoa, namely *B. hominis*, *E. coli*, *E. nana*, *G. lamblia*, and *I. buetschlii* were identified with a prevalence of 42.3% in this study area. The most frequent protozoa infection was *B. hominis* (21.3%), and the least encountered protozoa were *E. hystolitica* (0%) (Table 2). Among 169 positive individuals, the most of the students (35.9%) had single infection. Twenty-six (6.4%) of the students were infected with more than one intestinal protozoa, in which 23 (5.9%) had double intestinal protozoa infection and 3 (0.5%) had triple infections (Fig. 1). Among age groups, the most protozoa prevalence was reported among the age group of 13–15 yr old (*P*>0.05) (Table 1).

### Determinant possible risk factors

The results of the personal questionnaire were used to determine possible risk factors for IPIs prevalence. In univariate logistic regression analysis of risk factors potentially associated with protozoa infection prevalence (Table 3), significant relationship was noticed between intestinal protozoa infections with economic status, the water resources for drinking uses, and the methods of washing vegetables (*P*<0.05). Intestinal protozoa infections were independent of hand washing habit after toilet, parent’s educational status, occupation of parents, and grade of students (*P*>0.05).

## Discussion

This study aimed to assess the prevalence of protozoa infection and determinant factors among the schoolchildren of Sanandaj City. There is no previous study on the prevalence of protozoa infection in the study area.

This research found that the overall prevalence of intestinal protozoa infection was 42.3%; no case of helminthes infection was detected. It was a bit lower compared with other studies conducted among schoolchildren in other places. For example, the prevalence of intestinal protozoa infection was reported 95.95% in Ouagadougou region, Burkina Faso; 63.2% in Amalapuram, India; and 69.2% in North Gondar region of Ethiopia (16–18). Prevalence of protozoa infection was also relatively higher compared with other studies conducted on schoolchildren in Nakhon Ratchasina (17.3%) and in north India (36.7%) (19, 20). Such differences in prevalence could be attributed to different factors including seasonal and temporal variation during the implementation of the study, environmental conditions, socioeconomic status of parents and study subjects, and other geographical factors.

The result of the present study is also comparable with previous studies conducted in different parts of Iran. A high prevalence (50.57% and 47.7%) was recorded among schoolchildren in Urmia and at Khorasan province, Iran, respectively (21, 22). The lower prevalence in this study could be attributing to the habit of hand washing after toilet, educational level of parents, and occupation of parents. Nevertheless, the percentage of protozoa infections in the current study was higher than other studies reported in Gorgan (37.7%), in Mazandaran (29.7%), and in Yazd (9.5%) (23–25). This might be because of poor economic status, using well water for drinking purposes, and washing vegetables without applying disinfectants.

Totally, 169 students found positive for any protozoa infections, schoolchildren with single, double, and triple infections were 35.9, 5.9, and 0.5%, respectively. The prevalence of multiple infections was lower (11, 16). The present study indicated that multiple infections were higher than that reported in other studies (26). This difference could be because of different climatic conditions, sample size determination, as well as study time. The most frequent protozoa infection was by *B. hominis* followed by *E. coli* and *E. nana* infections with the prevalence of 21.3, 4.5, and 3.8%, respectively. The least encountered protozoa were *E. hystolitica*. This finding is similar with the results of Balcioglu and Ben-shimol, who reported that the most common protozoa were *B. hominis* with prevalence of 35 and 11.8% respectively (27, 28). Pathogenicity of *B. hominisis* is unclear; a recent study observed acute and chronic digestive disorders such as irritable bowel syndrome (IBS) (29). In general, the problem of protozoan infection is a chronic disease and most people showed asymptomatic then people had no awareness but it can cause chronic diarrhea (29, 30).

The prevalence rate of the intestinal protozoa infection is varied in different area (urban and rural). The difference in prevalence rate might be due to culture, living standard, and category of the study population. Difference due to gender was not observed in this study (*P*>0.05); although male (42.5%) had slightly higher prevalence rate compared with female (41.3%), which is the same as reported in another study (28). This could be because of more active and outdoor wandering nature of male children than female children (31). However, girls had a significantly higher prevalence of the intestinal parasites compared to boys (32). In the study, we observed that intensity of infection was higher in secondary school than primary school, but no statistically significant difference was observed among schools (*P*>0.05). Intestinal protozoa infections are more prevalent in the age groups 13–15 yr than the younger counterparts in the study area are, but there was no significant difference (*P*>0.05). This might be related to the high activities and behavior of children in this age group; they usually play and move around covering wider territory whereby the possibility of acquiring infections is increased. Nonetheless, the result was adverse with some of the reports, which showed a marginally higher positive rate among younger children (33, 34).

There was no case of helminthes infections in this study. It could be because of the public investments in basic sanitation, improvement of general living conditions, and the accessibility to health services, which this results are similar to the reports carried out in the parts of Iran (21, 35).

This research exhibited significant correlations between poor economic status and protozoa infections. Moreover, using water from the unprotected well was a risk factor for intestinal protozoa infection in schoolchildren (*P*<0.05). This may arise from the contamination of water with animals and human waste discharging into the wells (16). Based on the results of the univariate analysis, applying disinfectant for washing vegetables reduced the infection rate similar to a study in Tehran, Iran (12). Overall, 367 (91.75%) students had hand washing habit with water and soap after toilet and there was no significant difference between the rate of protozoa infection and hand washing after toilet (*P*>0.05). This might be due to high knowledge of children about the fecal-oral transmission of protozoa infection through their unwashed hands and relatively high rate of positive responses by the students and their parents (12). This is in contrary to the results of another research (36). In addition, with increased parent’s educational level, protozoa infections declined. However, no statistical difference was found (*P*>0.05) (12, 37, 38). Based on our findings, certain hygienic child-care practices were better when the parents had received a higher level of education. Children of farmers more likely exposed to the protozoa cysts compared other occupation of parents, but there was no significant difference (*P*>0.05). Moreover, no significant association was observed between grade level of students and rate of protozoa infections. This was in contrast to another study (36). The limitations of the study were the relatively low number of stool samples and low number of studied parasites species. It is necessary to develop control strategies including implementing of health education for students and their families and improving environmental sanitation.

## Conclusion

A relatively high prevalence of intestinal protozoa infections was revealed especially *B. hominis* among schoolchildren in Sanandaj City, while helminthes were no found. Thus, it is crucial to have prompt implementation of educational programs for students and their families. Physicians, school teachers and mass media should create awareness among school-children and their parents for effective prevention and control of protozoan infections in the study area.
